# An analog of glibenclamide selectively enhances autophagic degradation of misfolded α1-antitrypsin Z

**DOI:** 10.1371/journal.pone.0209748

**Published:** 2019-01-23

**Authors:** Yan Wang, Murat C. Cobanoglu, Jie Li, Tunda Hidvegi, Pamela Hale, Michael Ewing, Andrew S. Chu, Zhenwei Gong, Radhika Muzumdar, Stephen C. Pak, Gary A. Silverman, Ivet Bahar, David H. Perlmutter

**Affiliations:** 1 Department of Pediatrics, University of Pittsburgh School of Medicine, Pittsburgh, Pennsylvania, United States of America; 2 Department of Computational and Systems Biology, University of Pittsburgh School of Medicine, Pittsburgh, Pennsylvania, United States of America; 3 Department of Pediatrics, Washington University School of Medicine, St. Louis, Missouri, United States of America; Univerzitet u Beogradu, SERBIA

## Abstract

The classical form of α1-antitrypsin deficiency (ATD) is characterized by intracellular accumulation of the misfolded variant α1-antitrypsin Z (ATZ) and severe liver disease in some of the affected individuals. In this study, we investigated the possibility of discovering novel therapeutic agents that would reduce ATZ accumulation by interrogating a *C*. *elegans* model of ATD with high-content genome-wide RNAi screening and computational systems pharmacology strategies. The RNAi screening was utilized to identify genes that modify the intracellular accumulation of ATZ and a novel computational pipeline was developed to make high confidence predictions on repurposable drugs. This approach identified glibenclamide (GLB), a sulfonylurea drug that has been used broadly in clinical medicine as an oral hypoglycemic agent. Here we show that GLB promotes autophagic degradation of misfolded ATZ in mammalian cell line models of ATD. Furthermore, an analog of GLB reduces hepatic ATZ accumulation and hepatic fibrosis in a mouse model *in vivo* without affecting blood glucose or insulin levels. These results provide support for a drug discovery strategy using simple organisms as human disease models combined with genetic and computational screening methods. They also show that GLB and/or at least one of its analogs can be immediately tested to arrest the progression of human ATD liver disease.

## Introduction

ATD is a well-known genetic cause of severe liver disease including cirrhosis and hepatocellular carcinoma in adults. The classical form of ATD is characterized by a point mutation that substitutes lysine for glutamate 342 in the mutant variant called ATZ [1]. The substitution is known to favor misfolding of ATZ and sets up a kinetic-determined tendency for this variant protein to polymerize and form aggregates in the endoplasmic reticulum (ER) and perhaps other pre-Golgi vesicular compartments of the cell [2]. There is strong evidence that liver disease is caused by gain-of-function mechanisms triggered by the proteotoxic effects of misfolded ATZ accumulation. Genetic and environmental modifiers that target proteostasis mechanisms are hypothesized to account for wide variation in the hepatic phenotype among homozygotes for this disorder [1].

To identify modifiers of ATZ proteotoxicity, we used genome-wide RNAi screening of a *C*. *elegans* ATD model to identify genes that could increase or decrease the accumulation of ATZ [3]. We have previously shown that computational analysis of drug library screening using the *C*. *elegans* model can help identify novel therapeutic drug candidates [4]. In the present study, to identify other repurposable drugs, we developed a novel computational pipeline and this led to the identification of GLB as a candidate for reducing cellular ATZ accumulation. GLB is known to bind to members of the ATP-binding cassette (ABC) transporter family (subfamily C encoded by the gene ABCC8) for its mechanism of action, including most notably sulfonylurea receptor SUR1 that eventually leads to increased insulin secretion by pancreatic islet β-cells and mediates the therapeutic effect on blood glucose levels [5]. Although the link between ATP-dependent membrane transporters and intracellular ATZ accumulation was unclear we tested the effect of GLB on ATZ accumulation in mammalian cell line models. The series of experiments shows that GLB can reduce ATZ accumulation and provide structure-function relationships into the mechanism of drug action as well as new therapeutic strategies for preventing ATZ proteotoxicity.

## Results

### RNAi screening combined with computational systems pharmacology methods leads to GLB as a candidate repurposable drug for reducing ATZ accumulation

**Fig 1:**
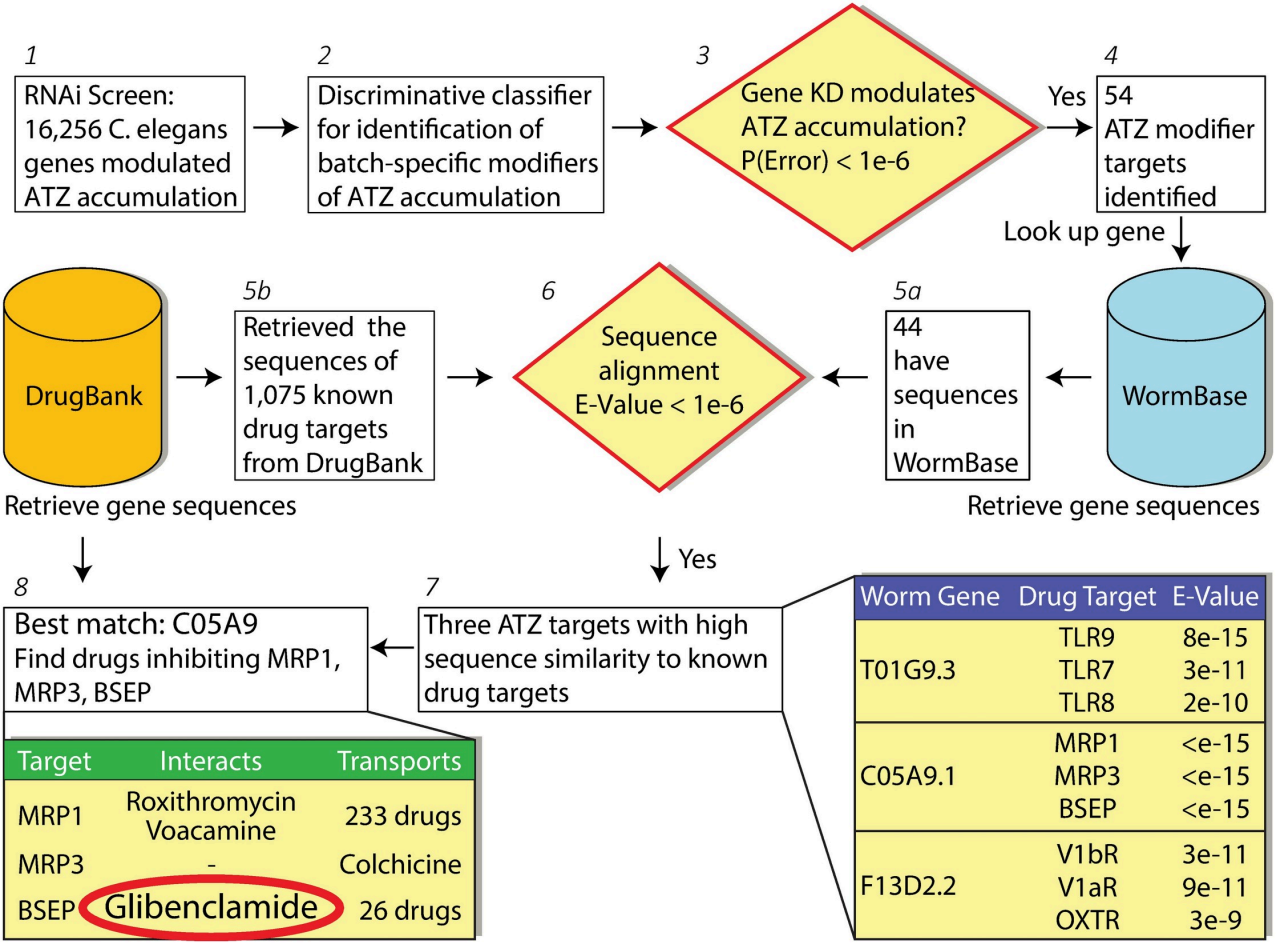
Computational systems pharmacology pipeline identifies GLB as a potential repurposing candidate. Computational workflow of eight steps (1–8) customized to assess repurposable drugs against ATD using RNAi screening data (step 1). Specifically we trained a discriminative logistic regression classifier to distinguish the modulators of ATZ accumulation (step 2–4). We compared the sequence of these genes to those of the known targets and identified three human orthologues that had significant sequence similarity to the known drug targets (steps 5–7). Of these, we picked the highest confidence gene (step 8) and identified the drug that interacts with the least promiscuous human targets based on available data, leading to GLB.

The computational systems pharmacology pipeline developed here consists of three parts (Fig 1): identification of ATZ modifier genes in the *C*. *elegans* model of ATD [3]; human target identification (i.e. human orthologue or homologue of the putative modifier gene from *C*. *elegans*; drug repurposing prediction. Out of 16,256 *C*. *elegans* genes we used a logistic regression based batch-specific hit calling method to identify 54 genes whose knockdown significantly modulated ATZ accumulation. We mapped 44 of these 54 genes to their human sequences using Worm Base [6]. Simultaneously we retrieved the sequences of the 1,075 targets of known, FDA-approved drugs from the DrugBankv3.0 [7]. Through sequence comparisons using BLAST, we identified three worm genes with high sequence similarity to known drug targets. Of these the gene C05A9.1 (the product of which is p-glycoprotein related 5; pgp-5) was found to exhibit the highest sequence similarity to three ATP-dependent membrane proteins that are targets of known drugs, ABC transporters subfamily C members 1 and 3 (ABCC1, ABCC3) and subfamily B member 11 (ABCB11, also known as bile salt export protein, BSEP). Of these ABCB11 had only one reported interaction in DrugBankv3.0, and that was GLB.

### GLB reduces ATZ levels in mammalian cell line models of ATD

**Fig 2:**
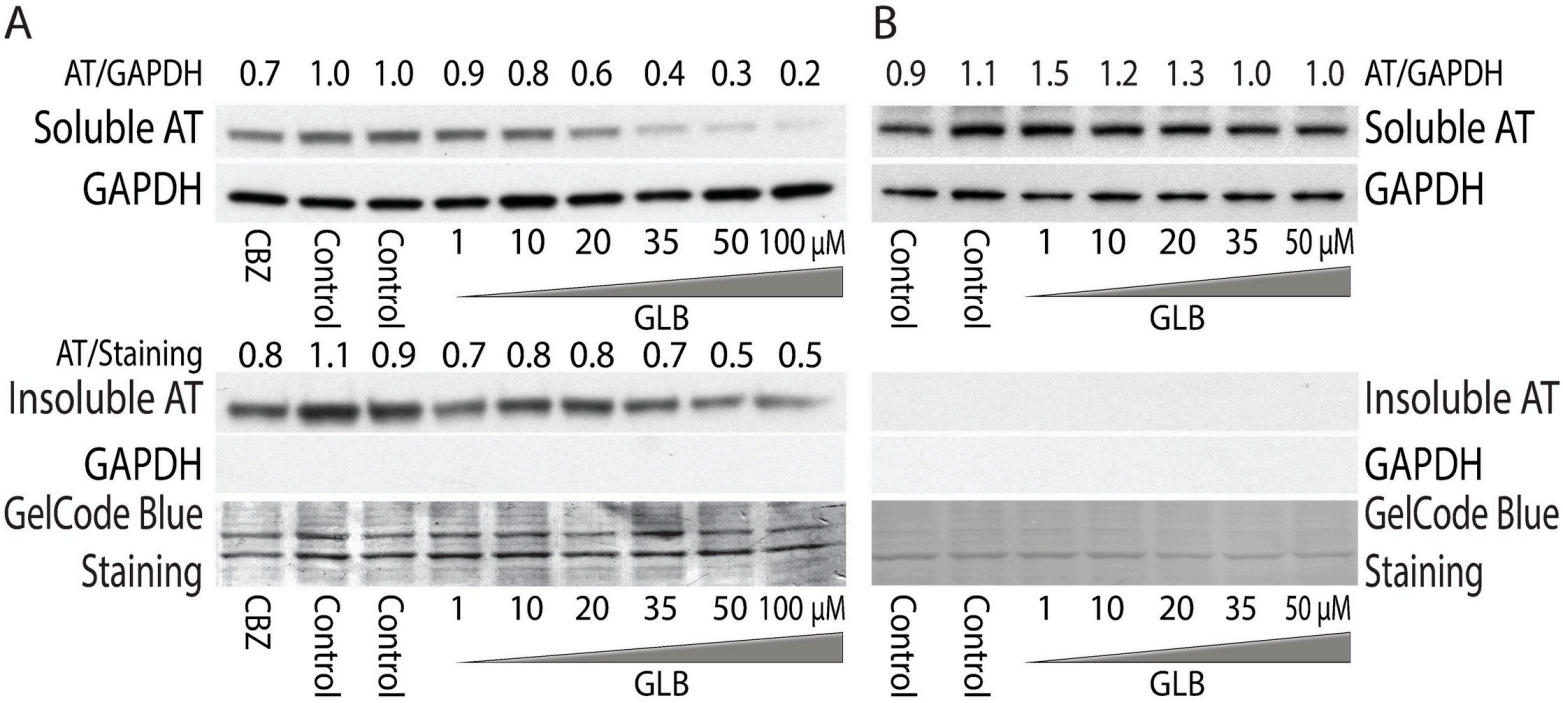
Effect of GLB on steady-state levels of ATZ in HTO/Z (A) and HTO/M (B) cell lines. Immunoblot analysis of cell lines treated with various concentrations of GLB. Carbamazepine at 30 μM was used as a positive control. After 48h incubation of GLB or CBZ, cells were harvested and separated into soluble and insoluble fractions for western blotting with anti-AT. GAPDH was used as a loading control for the soluble fraction and to validate the separation of soluble and insoluble fractions. Gel Code blue staining was used a loading control for the insoluble fraction. The relative densitometric values for ATZ level in experimental versus control conditions are shown at the top to demonstrate the magnitude of the effect.

First, we used a mammalian cell line model with inducible expression of ATZ to investigate the effects of GLB. The HeLa-based HTO/Z cell line was incubated for 48 h with GLB in a range of doses. Western blot analysis showed that GLB mediated a marked decrease in steady-state levels of ATZ in both the soluble and insoluble fractions (Fig 2A). The effect of GLB on ATZ levels was dose-dependent in range of 1 to 100 μM (Figure A.1 in S1 File) and comparable to the positive control carbamazepine (CBZ). The effect was time-dependent, greater at 48 than at 24 h incubation (Figure A.2 in S1 File). The effect of GLB was specific to the Z variant because there was no change in steady state levels of wild type AT in HTO/M cell line (Fig 2B) or of the AT Saar variant in the HTO/Saar cell line (Figure A.3 in S1 File). In addition, we also confirmed the effect of GLB in another cellular model of ATD (Figure A.4 in S1 File), HG2TONGZT cell line, human hepatoma cell line HepG2 engineered for Tet-On inducible expression of an ATZ-CFP chimeric protein. The effect of GLB on both soluble and insoluble ATZ is similar to what we have seen with autophagy enhancer drugs CBZ and fluphenazine [4, 8] and we have previously speculated that the effect on the soluble fraction involved autophagic clearance of soluble ATZ polymers.

### GLB reduces ATZ levels by increasing intracellular degradation of ATZ

**Fig 3:**
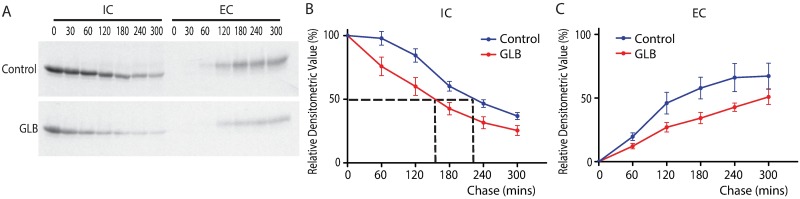
Effect of GLB on kinetics of ATZ secretion in HTO/Z cells by pulse-chase radiolabeling methods. (A) Fluorograms of control (top) and GLB-treated cells (bottom) using the HTO/Z cell line. IC = intracellular fraction; EC = extracellular fraction. After pulse-chase labeling, cell lysates and extracellular fluid were immunoprecipitated with anti-AT. (B) Densitometric analysis of kinetics for IC in the HTO/Z cell line. Kinetics of disappearance from IC was determined by densitometric analysis of fluorograms from five independent experiments. Dashed lines show the half-time for disappearance of ATZ in IC. (C) Densitometric analysis of kinetics for EC. Statistical analysis on kinetics utilized two-way ANOVA with Bonferroni correction (Mean ± SEM, n = 5).

To further characterize the effect of GLB on the kinetics of ATZ fate, we carried out ^35^S- methionine pulse-chase labeling experiments in HTO/Z cells. The cells were incubated for 48h in the absence or presence of GLB (40 μM), then were subjected to pulse radiolabeling for 60 mins and chase in medium with excess unlabeled methionine for time periods up to 300 mins. The results in Fig 3A showed that in the GLB-treated cells there is more rapid decay in labeled ATZ in the intracellular compartment (IC) at each time point of the chase period compared to the time 0, consistent with increased disappearance of intracellular ATZ. Further, less ATZ was detected in the extracellular compartment (EC) of the GLB-treated cells at every single time point of the chase period. These results are most consistent with a selective effect for the drug that accelerates intracellular degradation and leads to reduced intracellular accumulation of ATZ. Most importantly, quantitative analysis by densitometry on five independent experiments showed a statistically significant increase in disappearance of ATZ from the intracellular compartment in the cells treated with GLB with a half-time of 150 min compared with 220 min in control group (p < 0.0001) (Fig 3B). The rate of appearance and amount of ATZ in the extracellular fluid was decreased by GLB (p = 0.0001) (Fig 3C).

To characterize the effect of GLB on the synthesis of ATZ, we carried out pulse-labeling and real-time RT-PCR experiments in HTO/Z cell line. We found no difference at neither mRNA level (p = 0.582) or protein level (p = 0.9757) for ATZ comparing the control and GLB-treated cells (Figure B.1 and B.2 in S1 File). Therefore GLB had no effect on the synthesis of ATZ. Together these results suggest that the effect of GLB on ATZ is exclusively an increase in intracellular degradation. GLB had no effect on the kinetics of wild type AT (Figure B.3 in S1 File).

### The effect of GLB on ATZ degradation involves an autophagic mechanism

**Fig 4:**
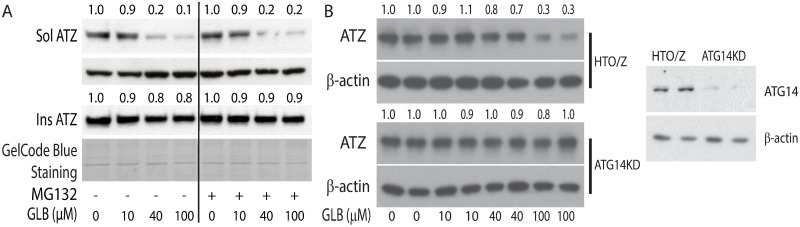
Mechanism of ATZ degradation targeted by GLB. (A) Effect of GLB on ATZ levels in the HTO/Z cell line in presence or absence of MG132. Cells were incubated for 48 hours with GLB (40 μM) and MG132 (30 μM) was added for the last 6 hours of this incubation period. Relative densitometric values are shown at the top. (B) Effect of GLB on ATZ levels in HTO/Z cell line and autophagy-deficient HTOZATG14KO (ATG14KO) subclone. Cells were incubated for 48 hours with varying doses of GLB (bottom) and then analyzed for ATZ and β-actin by immunoblotting. Blotting for ATG14 is shown on the right.

To begin to determine the mechanism by which GLB increases the rate of ATZ degradation we investigated the involvement of the proteasomal and autophagic pathways (Fig 4). The proteasomal inhibitor MG132 did not alter the effect of GLB on ATZ levels (Fig 4A) but in a subclone of the HTO/Z cell line rendered deficient in ATG14 the effect of GLB was almost completely abrogated (Fig 4B). Furthermore, the effect of GLB on ATZ levels was markedly diminished in the presence of lysosomotropic agents, ammonium chloride and chloroquine, and in the presence of bafilomycin, an inhibitor of the lysosomal V_0_ATPase that is part of the final stages of autophagy (Figure C in S1 File). Together, these data provide strong evidence that the autophagic pathway is the target of GLB action that mediates increased intracellular degradation of the misfolded ATZ variant. Parenthetically, it is interesting to note the effects of these lysosomotropic agents in the absence of GLB----greater accumulation of ATZ in the presence of bafilomycin (Figure C.3 and C.4 in S1 File) but not in the presence of ammonium chloride and chloroquine (Figure C.1, C2 and C.4 in S1 File), implicating some differences in mechanism of degradation of ATZ in baseline conditions as compared to conditions drugged by GLB.

To provide initial data on whether the drug also activates the autophagy pathway in a general way, we examined autophagy markers in the HTO/Z cell line by immunoblotting with anti-LC3 and anti-p62. The results from HTO/Z cells showed increased LC3-II to LC-I ratio and decreased p62 level in GLB-treated cells (Figure D.1 in S1 File) GLB also increased the LC3-II to LC3-I ratio in the HG2TONGZT#1 cell line (Figure D.2 in S1 File). These results provide initial suggestive but not definitive evidence that GLB has an activating effect on autophagy.

To further characterize the autophagic pathway that is targeted by GLB, we carried out 2 other series of experiments. First, we tested the effect of GLB in a subclone of the HTO/Z cell line deficient in ATG5. The HTO/Z cell line was transfected with shRNA constructs for ATG5 and the resulting ATG5 knockdown cell lines (HTOZATG5KD) were selected in 2 μg/ml puromycin for two weeks. Immunoblotting with anti-ATG5 and anti-LC3 showed that the HTOZATG5KD cells have markedly decreased ATG5 level and decreased LC3-II to LC3-I ratio (Figure E.1 in S1 File). Interestingly, the effect of GLB in reducing ATZ levels was maintained in the HTOZATG5KD cell line (Figure E.1 in S1 File). The effect of GLB on ATZ levels in the HTOZATG5KD cell line was blocked by bafilomycin (Figure E.2 in S1 File), providing validation for this subclone as an appropriate model. Thus, ATG14 is required for the mechanism of GLB on ATZ degradation but ATG5 does not appear to play a major role in the autophagic mechanism.

Second, to determine whether GLB induces autophagy through the mTOR-depedent signaling pathway, we examined the phosphorylation of the mTOR substrates: p70 S6K and 4E-BP1. HTO/Z cells were incubated in presence or absence of GLB and then cell homogenates harvested in NP-40 buffer containing phosphatase inhibitors were investigated for total and phosphorylated proteins by immunoblotting (Figure F.1 in S1 File). Torin-1 at 250nM was included as a positive control which markedly de-phosphorylated mTOR substrates as expected. In contrast GLB had no effect on phosphorylation of p70 S6K or 4E-BP1, indicating that effect of GLB on autophagy is independent of the classic mTOR signaling pathway (Figure F.1 in S1 File). We did not detect any effect of GLB on downstream targets of the unfolded protein response (UPR) (Figure F.2 in S1 File) and thus we concluded its effect is also independent of the UPR.

### Potential targets for the action of GLB on autophagic degradation of ATZ

**Fig 5:**
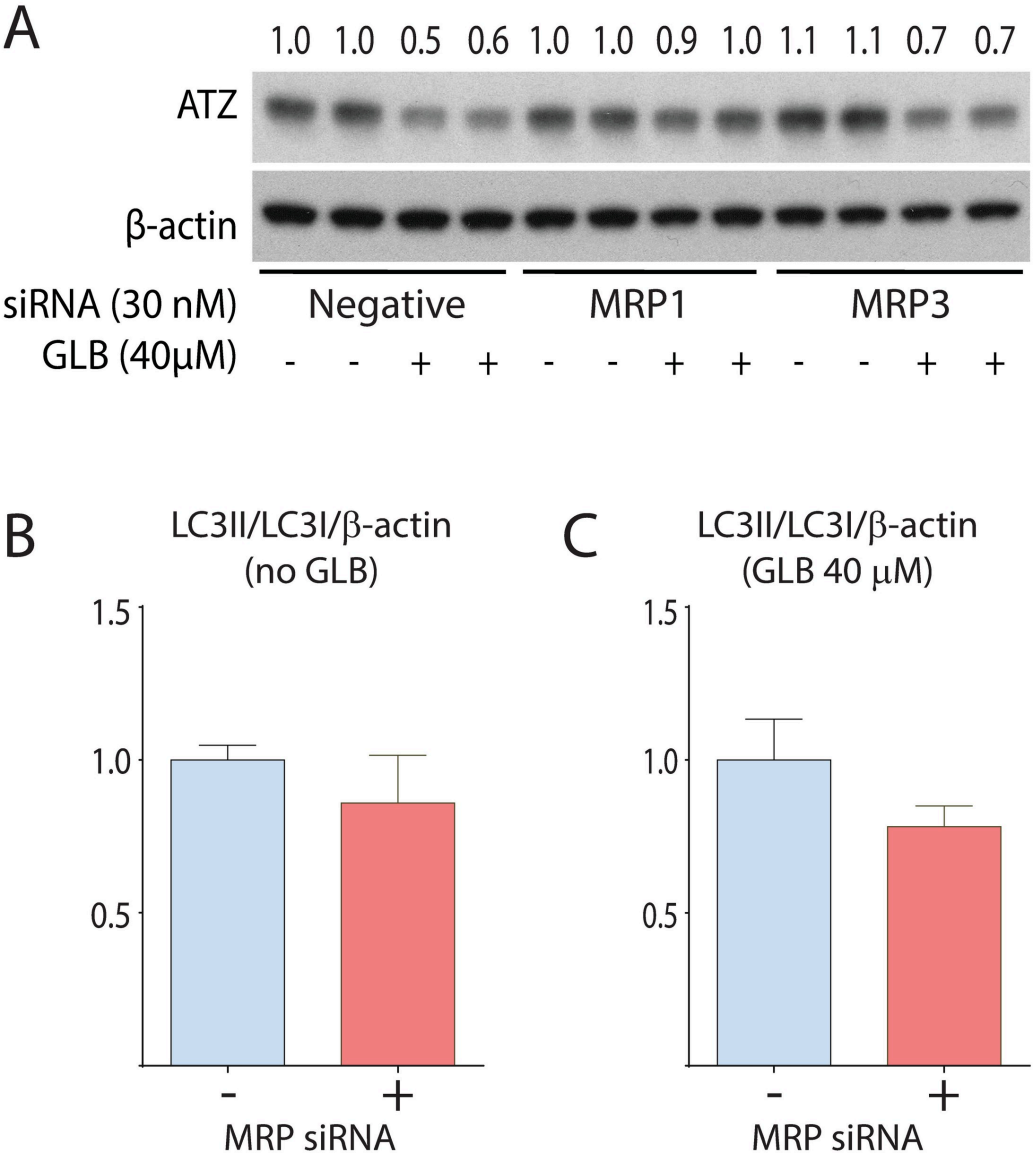
Effect of GLB on ATZ levels in the HTO/Z cell line in the absence or presence of MRP1 or MRP3 siRNA. HTO/Z cells were transfected with MRP1 siRNA, MRP3 siRNA or negative control siRNA at a final concentration of 30nM. After transfection, the cells were incubated in the presence or absence of GLB for 48 hours. Immunoblotting analysis for ATZ and β-actin is shown in A. Effect on LC3-II/LC3-I/β-actin ratio in control cells is shown in B and in GLB-treated cells in C, using densitometric scanning and arbitrarily setting the value in the absence of MRP siRNA at a value of 1.0.

To potentially address the question of whether GLB acts on autophagic degradation of ATZ through an off-target effect, we considered several possible targets. SUR1 was considered an unlikely target because GLB acts on it in nanomolar doses [9] whereas doses in the micromolar range were required for the action of GLB on ATZ degradation in the HTO/Z cell line. Next we considered the ABC transporter family identified by the combined RNAi/computational screening that led to GLB as a candidate therapeutic (Fig 1). We excluded BSEP (ABCC11) because it is not expressed in HeLa cells. Thus, we investigated the possibility that one or both of the other relatively closely related ABC transporters, MRP1 (ABCC1) and MRP3 (ABCC3), which are also known to be targets of GLB binding and drug action [10, 11] mediates the effect on autophagic degradation of ATZ. We used siRNA to knockdown expression of MRP1 and MRP3 in the HTO/Z cell line (Fig 5; Figure G in S1 File). The results show a significant blocking of the GLB effect on ATZ levels in the HTO/Z cells rendered deficient in MRP1 but not for MRP3 (Fig 5A). MRP1 siRNA had no effect on the LC3-II to LC3-I ratio in control or GLB-treated cells (Fig 5B and 5C), suggesting that it inhibits drug action rather than an independent effect on autophagy. Although these results do not completely account for the target of GLB action on autophagic degradation of ATZ, they do suggest that at least one member of the ABC transporter family plays a role and that it is at least partially due to an on-target effect. These results also provide some additional confirmatory support for identification of pharmacological targets using combined RNAi/computational systems pharmacology screening as a discovery campaign.

### GLB analogs that reduce ATZ levels but do not induce insulin secretion in mammalian cell line models

**Fig 6:**
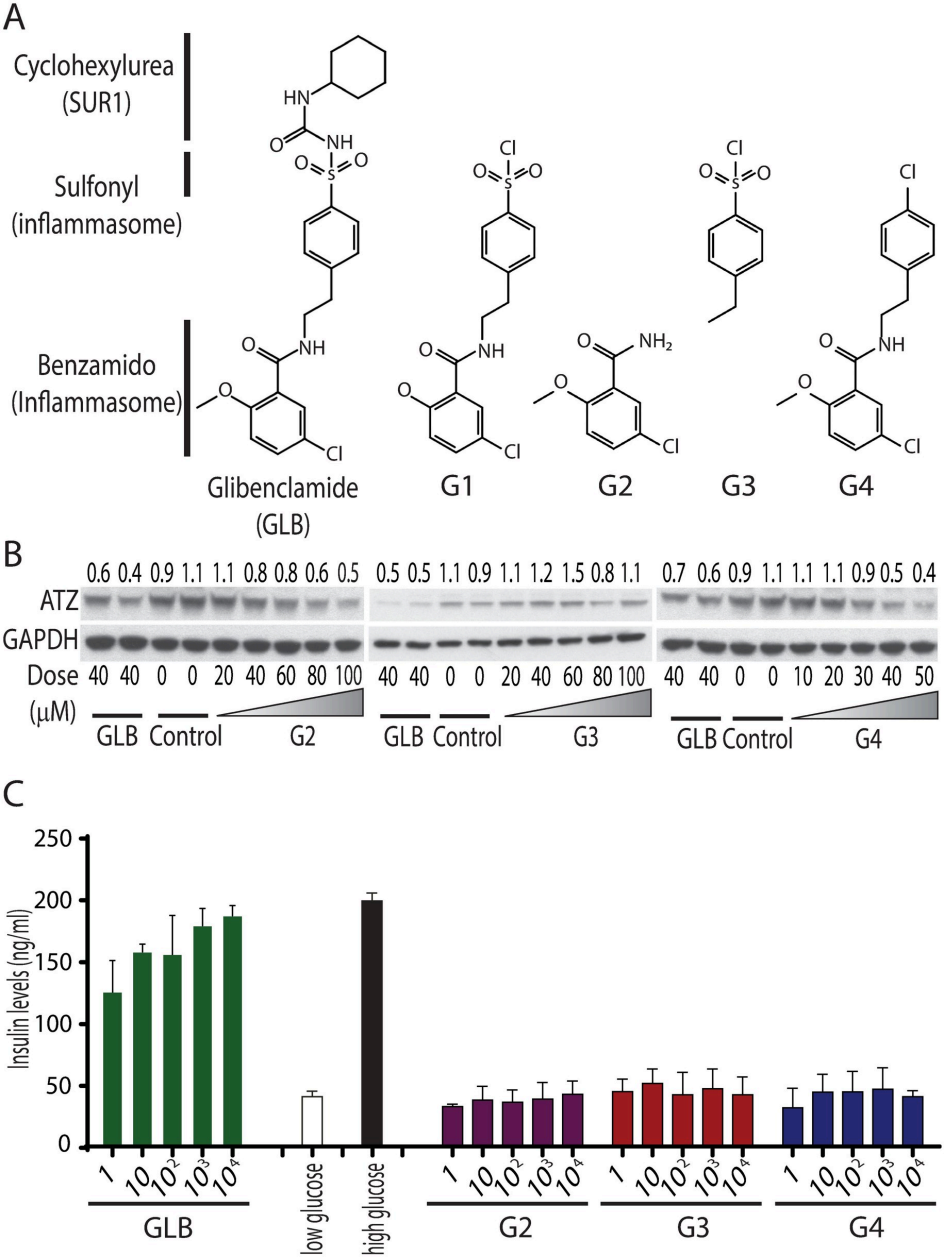
Effect of GLB analogs on ATZ levels and insulin secretion in mammalian cell line models. (A) The structures of GLB and GLB analogs. (B) Western blotting analysis of the effect of G2, G3 and G4 on ATZ levels in HTO/Z cell line. GAPDH was included as a loading control. GLB at 40 μM was used as positive control; DMSO was used as negative control. (C) Insulin levels in Min-6 pancreatic β-cell line. The cells were incubated with GLB, G2, G3 and G4 in the indicated doses. Low and high glucose conditions were used as negative and positive controls (Mean ± SD, n = 3). The results show that insulin secretion is significantly increased by GLB at doses as low as 1 nM (p<0.0001) but not affected by analogs G2 (p = 0.8483), G3 (p = 0.9222) or G4 (p = 0.7886).

Our results indicated that the micromolar doses of GLB were needed for its effect on ATZ disposal (Figs 2–5) whereas this drug increases insulin secretion at doses in the nanomolar range [9]. A study by Lamkanfi et al showed that GLB inhibits the inflammasome at micromolar doses [12] and, interestingly, its effect on the inflammasome could be reproduced by analogs of GLB that lacked the cyclohexylurea group reputed to be the domain of GLB that binds to the sulfonylurea receptor SUR1 (Fig 6A). These authors concluded that the cyclohexylurea group and binding to SUR1 is dispensable for the inflammasome inhibitory activity of GLB and, furthermore, speculated that the effect on the inflammasome was mediated by a member of the sulfonylurea receptor family distinct from SUR1. These results led us to investigate the effect of GLB analogs on ATZ disposal in the HTO/Z cell line model in comparison to their effects on insulin secretion in a pancreatic β-cell model with the hypothesis that the effect on ATZ disposal would be pharmacologically similar to the effect on the inflammasome. Three analogs that lack the SUR1-binding domain, G2, G3 and G4, were utilized (Fig 6A). The results in Fig 6B show that analogs G2 and G4 mediate dose-dependent reduction in steady state levels of ATZ very similar to that effect of parent GLB but G3 has a negligible effect on ATZ levels. Thus, the benzamido group of the GLB parent structure is essential for the effect on ATZ disposal. We also found that several additional analogs based on the structure of G4 elicit increased ATZ disposal (Figure H in S1 File), providing further confirmation of the importance of the benzamido group. Next we tested these analogs for insulin secretion using the Min6 pancreatic β-cell line (Fig 6C). Low glucose and high glucose conditions were included as negative or positive controls to validate this assay. The results showed that GLB induced insulin secretion (at doses as low as 1nM), but the GLB analogs G2, G3 and G4 had no effect at any dose. These results suggest that the benzamido group on the GLB parent structure is important for the effect on ATZ disposal just as it appears to be for inflammasome inhibition and is distinct from how GLB is recognized for its effect on insulin secretion. The results also provide further evidence for the conclusion that GLB does not act on autophagy through the SUR1 target. The effect of GLB on ATZ disposal may also differ from its effect on the inflammasome, especially as we did not see an effect of G3 on ATZ disposal here whereas it did mediate inhibition of the inflammasome in the previous report [12]. Nevertheless one of the important aspects of these results is the possibility that an analog of GLB, such as G2 and G4, could be used therapeutically to mediate ATZ disposal for ATD liver disease without having a hypoglycemic effect.

### GLB analog G2 reduces hepatic ATZ load and hepatic fibrosis in the PiZ mouse model in vivo without increasing blood glucose or insulin levels

**Fig 7:**
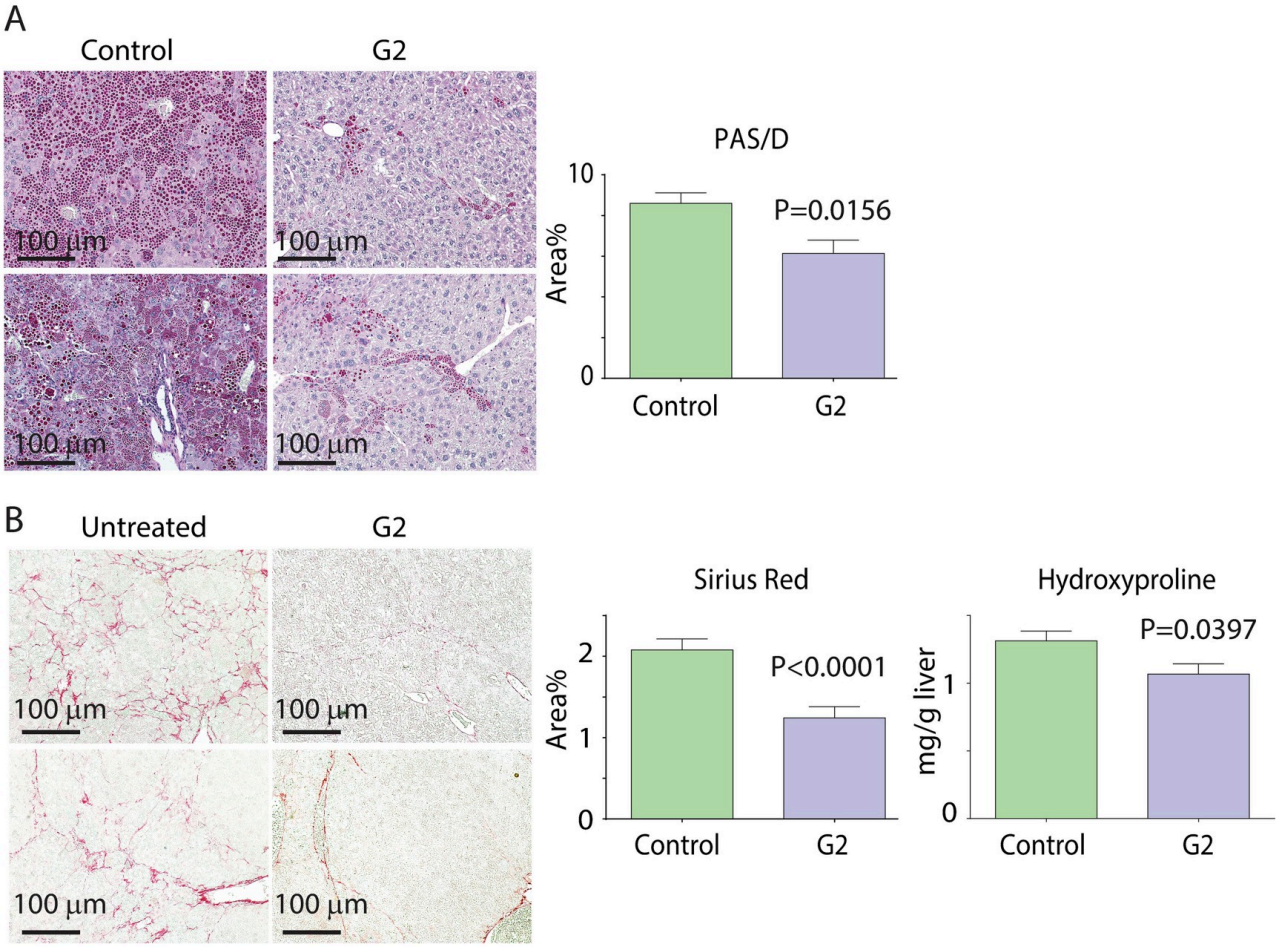
*In vivo* effect of GLB analog G2 on hepatic ATZ load (A) and hepatic fibrosis (B) in the PiZ mouse model. The drug was administered by orogastric gavage or subcutaneous osmotic pump predicted to deliver a dose of 10 mg/kg/day over 3 weeks to male PiZ mice at 4–6 months of age. A separate group of age-matched male PiZ littermates were treated with placebo by the same route of administration. Examples of PAS/D and Sirius red staining for livers from 2 untreated (left) and G2 treated mice (right) are shown in (A) and (B), respectively. Results of quantitative morphometric analysis from 4 separate experiments for PAS/D (n = 17, p = 0.0156) are shown on the right in (A). Results of quantitative morphometric analysis from 4 separate experiments for Sirius red (n = 23, p<0.0001) are shown on the right in (B). Results of hydroxyproline content analysis for 1 experiment (n = 7, p = 0.0397) is shown on the far right in (B). Statistical analysis used Unpaired, two-tailed Student t-test for PAS/D staining and hydroxyproline content and unpaired, two-tailed Student t-test with Welch-correction for the Sirius red staining data.

Because the G2 analog represents the minimal structural requirement for eliciting ATZ disposal at doses that did not affect insulin secretion in mammalian cell line model systems, it was selected for *in vivo* testing in the PiZ mouse model of ATD. The drug was administered by orogastric gavage or subcutaneous osmotic pump predicted to deliver a dose of 10mg/kg/day over 3 weeks to male PiZ mice at 4–6 months of age. A separate group of age-matched male PiZ littermates received a pellet with placebo (Fig 7). The drug and placebo were well tolerated and drug led to a statistically significant decrease in ATZ load as determined by PAS/D stained inclusions (Fig 7A) and by immunoblot analysis of steady state, total extractable ATZ levels (Figure I.2 in S1 File) and decreased fibrosis as determined by Sirius Red staining (Fig 7B). Another metric of fibrosis, hepatic hydroxyproline content was also significantly decreased in the mice treated with G2 (Fig 7C). Serum ATZ levels did not change significantly (post 12.48+/-1.41 compared to pre 13.20 +/- 1.20 in experimental group and compared to post 12.52+/-1.58 and pre 11.33+/-1.46 ng/ml in control group). Hepatic ATZ RNA levels were not affected by G2 treatment (Figure I.1 in S1 File). Steady state levels of p62 significantly declined in the liver of G2-treated PiZ mice (Figure I.3 in S1 File), suggesting that the drug increased autophagic activity *in vivo*. There were no significant changes in blood glucose or blood insulin levels. Blood glucose levels on day 7 were 91.86 +/- 10.38 mg/dl for the G2-treated mice (n = 7) versus 91.50 +/-15.40 for the placebo group (n = 7) and blood insulin levels were 0.3063 +/- 0.2156 ng/ml for the G2-treated mice (n = 6) versus 0.3138 +/-0.1562 for the placebo group (n = 6). There were no signs of toxicity, including serum levels of AST and ALT (AST: placebo = 140.4 +/- 22.7 vs. treated = 127.8 +/-19.43, NS; ALT: placebo = 66.1 +/- 13.7 vs. treated = 62.4 +/- 13.0, NS).

## Discussion

These results provide powerful validation for computer-aided drug discovery using simple organisms to model human diseases. The *C*. *elegans* model used here was genetically engineered for adaptation to high throughput, live animal screening techniques [13]. This platform and existing genetic and informatics tools were then used for genome-wide RNAi screening combined with computational analysis to generate a list of candidate drugs, and in particular GLB as a top-ranking candidate. Analysis of the effect of GLB in mammalian cell line models of ATD showed that its effect was exclusively on intracellular disposal of the misfolded ATZ variant. It is important to point out that the screening platform is designed to detect drugs which lower the cellular load of ATZ and this means that it could theoretically detect drugs which lower ATZ levels by effects on synthesis, degradation and/or secretion [13]. Yet the two drugs that have been identified by this platform and subjected to detailed analysis so far, fluphenazine [5] and GLB both target the degradative mechanism exclusively and this was also the case for carbamazepine [8].

As was the case for fluphenazine and carbamazepine, our data reported here on GLB suggest that its mechanism of action for increasing ATZ degradation is through the autophagolysosomal system. Its effect was blocked by bafilomycin and lysosomotropic agents and it was also shown to activate the autophagic response, including increased LC3-II to LC3-I ratio together with reduced p62 levels in the cell line model and in the livers of the PiZ mice *in vivo*. However, it may involve a somewhat unique non-canonical autophagy mechanism that is dependent on ATG14 but independent of mTOR. Because ATG14 has been implicated at very early and late stages of the conventional autophagy pathway, these results could mean that GLB acts on a mechanism that is distinct but converges with the canonical pathway at either early or late stages. Divergence from the conventional pathways for autophagy has emerged in an increasing number of recent studies [14].

The specific targets and mechanism of action of GLB on intracellular ATZ disposal will require further investigation. GLB was identified by the combined RNAi/computational screening campaign because it is known to bind to and inhibit members of the ABC transporter family, including MRP1, MRP3 and BSEP [10, 11]. Our results suggest that at least one of the members of this family, MRP1, is at least partially targeted by the mechanism of action. To our knowledge and review of the literature, MRP1 and other ABC transporters have not been previously implicated in regulation of autophagy. However there is some evidence that MRP1 influences intracellular calcium [15] and we know that the activity of the autophagy pathway is regulated by cellular calcium [16, 17].

GLB appears to be an ideal candidate for treatment of ATD patients predisposed to liver disease and perhaps for other diseases caused by the proteotoxic effects of misfolded proteins. In addition to enhancing intracellular disposal of misfolded ATZ it has been shown to inhibit the inflammasome and so it may have the additional benefit of preventing hyper-inflammation. Although it is entirely possible that patients will be able to tolerate potential for hypoglycemic effects or counteract these by simple dietary adjustments, the results reported here suggest that it will ultimately be possible to utilize analogs of the parent GLB structure that minimize effects on insulin secretion and blood glucose levels. These analogs may be particularly important because insulin signaling appears to impair the proteostatic response in model systems of several misfolded protein diseases, including ATD [18] and Alzheimer’s disease [19]. Two other important features are the palatability and the wide margin of safety for this drug. GLB is significantly more palatable than previously identified autophagy enhancer drug candidates. Carbamazepine and phenothiazines are notable for their sedative and dysphoric effects. We know that many patients with ATD completely escape liver disease and others have mild liver disease that does not affect their health in any substantive way but it is not yet possible to prospective differentiate these so-called ‘protected’ individuals. A drug with such a wide margin of safety could be used prophylactically to address this conundrum and perhaps also avert the clinical situations in which ATD is first diagnosed when end-stage liver disease is already irreversible. Furthermore, with a wide margin of safety, it could be used in a preventive paradigm targeting autophagy to slow age-dependent degeneration.

## Materials and methods

### Cell lines

HTO/Z, HTO/M, and HTO/Saar are human epidermal HeLa cell lines (Clontech #631156) with doxycycline (dox) -regulated expression of ATZ mutant, wild type AT, and AT Saar variant, respectively, as previously described [8]. The HG2TONGZT cell line is a human hepatoma cell line HepG2 engineered for Tet-On (Clontech # 631150) inducible expression of ATZ-CFP (cyan-fluorescent protein) chimera. The cellular properties of ATZ-CFP appear to recapitulate what is seen for ATZ in other genetically engineered cell lines, as previously described [4]. ATG5 knockdown cell lines (HTOZATG5KD) were created by transfecting HTO/Z cells with shRNA constructs (OriGene, Rockville, MD). The cells were selected in 2 μg/ml puromycin for two weeks and followed by further analysis. ATG14 knockout cell lines (HTOZATG14KO) were created by gene editing in the Genome Engineering and iPS Center in the Department of Genetics of Washington University School of Medicine.

### Drug treatment

For experiments with GLB, the Tet-off inducible cell lines were cultured in absence of doxycycline for at least 12 days to ensure expression of AT or its variants. The Tet-on inducible cell lines were cultured in the presence of doxycycline 200 ng/ml for 3 days for optimal expression of ATZ. The cells were then subcultured into separate monolayers in fresh complete growth medium and incubated for 48 hours in the absence or presence of GLB.

### Transgenic mice

PiZ mouse model has been described previously [8]. For experiments involving drug administration in mice in vivo, doses of 5–50 mg/kg/day for GLB for 3 weeks, and then liver histological and biochemical characteristics assessed exactly as previously described [8]. Each experimental group contained 4–6 four- to six-month old male mice randomly chosen from littermates. GLB and its analogs were delivered by oral gavage or slow-release subcutaneous pellets formulated by Innovative Research of America. Control mice were littermates given DMSO by gavage or placebo pellets specifically designed for each of these drugs. The outcome was determined by evaluating 1) the hepatic ATZ load by immunoblotting with anti-AT, immunostaining with PAS/D; 2) hepatic fibrosis using Sirius Red staining; 3) autophagy by immunoblotting with anti-LC3 and anti-p62, and imaging for GFP+ autophagosomes; 4) monitoring blood glucose levels by glucometer and blood insulin levels by ELISA Wide Range Assay (CrystalChem). For immunostaining, liver samples were fixed in 10% Formalin, followed by staining with PAS after diastase and Sirius Red using standard techniques [8]. Quantitative evaluation of immunostaining and histochemical staining was carried out by a member of the team that was blinded to group allocation. Hydroxyproline content was determined by an enzyme-based microplate colorimetric assay (Sigma).

### Insulin secretion assay

Pancreatic β cells Min6 were maintained in DMEM high glucose with the supplemental of 10% FBS, 1% penicillin/streptomycin and 5 μl/L 2-mercaptoethanol. Insulin secretion assays were performed using insulin ELISA kits from Crystal Chem INC (Downers Grove, IL). Briefly, cells were plated in 24 well plates 48-hour before the assay. The cells were treated with increasing doses of GLB or the analogs ranging from 1 nM to 10 μM. On the day of experiments cells were washed twice and incubated with Krebs-ringer bicarbonate buffer (KRBB) containing 2.5 mM glucose and 0.1% BSA for 1 hour. Buffer was removed from the cells and fresh KRBB containing chemicals (GLB or analogs) or 16 mM glucose (used as positive control) were added to the cells and incubated for 1 hour. The supernatant was collected right after the incubation and insulin concentration was assessed according to manufacturer’s instructions.

Materials and methods for genomic engineering of cell lines, siRNA experiments, immunoblot analysis, radio-immunoprecipitation/SDS-PAGE, real-time quantitative PCR and statistical analysis are described in Supplementary Materials.

### Animal committee approvals

All animal experiments were approved by the IACUC at University of Pittsburgh and the ASC of Washington University in St. Louis.

## Supporting information

S1 File Supporting information file (S1) including methods and nine supporting figures. **Figure A**. Effect of GLB on steady state levels of AT variants in mammalian cell line models. **Figure B**. Effect of GLB on synthesis of ATZ in HTO/Z cell line, RNA levels in HTO/Z cell line and on kinetics of secretion of AT in HTO/M cell line. **Figure C**. Mechanism of ATZ degradation targeted by GLB. **Figure D**. Effect of GLB on LC3 conversion and p62 levels. **Figure E**. Effect of GLB on ATZ levels in the ATG5-deficient HTOZATG5KD cell line. **Figure F**. Effect of GLB on phosphorylation of TOR substrates (p70, S6K and 4E-BP1) and unfolded protein response in HTO/Z cell line. **Figure G**. Effect of MRP1 and MRP3 and negative control siRNA on MRP1 and MRP3 mRNA levels in HTO/Z cell line. **Figure H**. Effect of G4 analogs (G5, G8 and G9) on ATZ. **Figure I**. In vivo effect of GLB analog G2 on hepatic ATZ and p62 levels.
(PDF)
