# Supplementary material for: An analog of glibenclamide selectively enhances autophagic degradation of misfolded α1-antitrypsin Z
Source: PLoS One. 2019 Jan 23;14(1):e0209748. doi: 10.1371/journal.pone.0209748 (PMC6343872; doi:10.1371/journal.pone.0209748)
Supplement: S1 File — Figure A. Effect of GLB on steady state levels of AT variants in mammalian cell line models. Figure B. Effect of GLB on synthesis of ATZ in HTO/Z cell line, RNA levels in HTO/Z cell line and on kinetics of secretion of AT in HTO/M cell line. Figure C. Mechanism of ATZ degradation targeted by GLB. Figure D. Effect of GLB on LC3 conversion and p62 levels. Figure E. Effect of GLB on ATZ levels in the ATG5-deficient HTOZATG5KD cell line. Figure F. Effect of GLB on phosphorylation of TOR substrates (p70, S6K and 4E-BP1) and unfolded protein response in HTO/Z cell line. Figure G. Effect of MRP1 and MRP3 and negative control siRNA on MRP1 and MRP3 mRNA levels in HTO/Z cell line. Figure H. Effect of G4 analogs (G5, G8 and G9) on ATZ. Figure I. In vivo effect of GLB analog G2 on hepatic ATZ and p62 levels. (PDF) [file pone.0209748.s001.pdf]

Supporting Information

An analog of glibenclamide enhances autophagic degradation of misfolded α1-antitrypsin Z variant without affecting insulin secretion

Authors: Yan Wang [1], Murat C. Cobanoglu [2], Jie Li [1,3], Tunda Hidvegi [1,3], Pamela Hale [1,3], Michael Ewing [1], Andrew S. Chu [1], Zhenwei Gong [1], Radhika Muzumdar [1], Stephen C. Pak [1,3], Gary A. Silverman [1,3], Ivet Bahar [2], David H. Perlmutter [1,3] *

Affiliations:

[1] Department of Pediatrics, University of Pittsburgh School of Medicine, Pittsburgh, PA, USA

[2] Department of Computational and Systems Biology, University of Pittsburgh School of Medicine, Pittsburgh, PA, USA

[3] Department of Pediatrics, Washington University School of Medicine, St Louis, Missouri, USA

* Correspondence should be addressed to DHP (perlmutterd@wustl.edu)

Methods

Materials. Goat anti-human AT was purchased from Diasorin (Stillwater, MN, #80252). Antibody to LC3 and GAPDH was purchased from Sigma (St. Louis, MO; L7543, G8795). Antibody to p62 (#51145), ATG5 (#12994), and mTOR substrate antibody sampler (p46-BP1: #2855; 4E-BP1: #9644, Phospho-p70 S6K: #9234, S6K: #2708) were purchased from Cell Signaling Technology (Danvers, MA). Glibenclamide (GLB) was purchased from TOCRIS and all GLB analogs were from Enamine (Monmouth Jct., NJ) or Ryan Scientific (Mt Pleasant, SC); all chemicals were prepared in a stock solution of 100 mM in DMSO. Carbamazepine (CBZ) was purchased from Sigma and prepared in a stock solution of 25 mg/ml DMSO. Doxycycline was purchased from Sigma and prepared 1mg/ml in water. Glibenclamide (GLB) in sustained release pellets was manufactured by Innovative Research of American (Sarasota, FL). MG132 was purchased from Calbiochem (stock solution 10 mM in DMSO), E64D from Peptide International (stock solution 20 mg/ml in DMSO) and pepstatin A from Sigma (stock solution 20 mg/ml in DMSO).

Genomic engineering of cell lines. For targeted disruption of ATG14 in the HTO/Z cell line, *ATG14* was targeted at exon 5 with guide RNA 5′-AAGAAGTCATTATGAGCGTC-3' cloned into the MLM3636 gRNA expression vector (gift from Keith Joung, Addgene plasmid #43860) as previously described (1). HTO/Z cells (1 x 10^6) were transfected using 500 ng guide RNA plasmid and 2 ug of Cas9 expression plasmid (gift from Jin-Soo Kim, Addgene plasmid #43945) via nucleofection using 100 ul of solution P3 and program CN-114 (Lonza 4D-Nucleofector, X unit). Transfected pools were single cell sorted into 96-well plates using a MoFlo Cell Sorter and expanded for 2-3 weeks prior to harvesting. Targeted deep-sequencing was performed to determine genotype and clone purity using target specific primers forward 5' TCTGAAGGCCTTCTCAAAACCAAGGA 3' and reverse 5' TCTGCGGTGCGTACTGTTTGTTGAA 3'. PCR was used to confirm the presence of the ATZ (forward 5' ACCCGGGTCGAGTAGGCGTGTA 3' and reverse 5' GCTGGCTGTCTGGCTGGTTGA 3') and TET (forward 5'CATTGACGCAAATGGGCGGTAG and reverse 5' GCCAATACAATGTAGGCTGCTCTAC 3') expression plasmids for each clone. All PCR amplifications were performed with MyTaq Red Mix (Bioline), according to the manufacturer protocol. Cell lines were tested for mycoplasma contamination.

SiRNAs. ON-TARGETplus SMART pool siRNA for MRP1 (Dharmacon: L-007308-00) and MRP3 (Dharmacon: L-007312-00) were used for the investigation of ABC transporters. ON-TARGETplus Non-targeting Pool was used as a control (Dharmacon: D-001810-01-20). The HTOZ cells were transfected at a final concentration of 30nM with Lipofectamine 2000 (Thermo Fisher Scientific). The cells were incubated for 48 hours in the presence or absence of GLB (40μM) after the transfection. Samples were subjected to real-time RT-PCR for MRP1 and MRP3 mRNA expression and then immunoblot analysis for ATZ and β-actin.

Proteasome inhibitors and LC3 immunoassays. For experiments in which proteasomal inhibitors were used, MG132 was used at 30 μM for the last 6 hours of the incubation with GLB or control. Cells that were incubated with MG132 alone served as control to validate that the proteasome was inhibited.

For investigation of LC3 conversion, lysosomal inhibitors (E64D and pepstatin A at 20 μg/ml) were added to the medium for the last 4 hours (or overnight) of the incubation with GLB or control. This has been shown to inhibit the lysosomal degradation of LC3-II and when compared to the LC3-II levels in the absence of lysosomal inhibitors to provide a reflection of autophagic flux. Other lysosomal inhibitors include chloroquine (CLQ) at 50μM, ammonium chloride at 10 mM, used in overnight incubation time periods. Bafilomycin was used at a concentration of 100 nM in overnight incubation trials.

Insulin Secretion Assay. Pancreatic β cells Min6 were maintained in DMEM high glucose with the supplemental of 10% FBS, 1% penicillin/streptomycin and 5 μl/L 2-mercaptoethanol. Insulin secretion assays were performed using insulin ELISA kits from Crystal Chem INC (Downers Grove, IL). Briefly, cells were plated in 24 well plates 48-hour before the assay. The cells were treated with increasing doses of GLB or the analogs ranging from 1 nM to 10 μM. On the day of experiments cells were washed twice and incubated with Krebs-ringer bicarbonate buffer (KRBB) containing 2.5 mM glucose and 0.1% BSA for 1 hour. Buffer was removed from the cells and fresh KRBB containing chemicals (GLB or analogs) or 16 mM glucose (used as positive control) were added to the cells and incubated for 1 hour. The supernatant was collected right after the incubation and insulin concentration was assessed according to manufacturer's instructions.

Immunoblots. **C**ells were lysed in NP40 buffer, and then separated into insoluble and soluble fractions according to our previously established technique (2, 3). Samples were subjected to immunoblot analysis for AT and GAPDH. For immunoblot analysis to detect AT or GAPDH, cells were lysed in 50 mM Tris-HCl, 150 mM NaCl, 1% NP-40, pH 8.0. Protein concentrations were determined by BCA protein assay (Pierce Biotechnology, Rockford, IL). Samples (10-40 μg) were boiled for 5 min and electrophoresed on a 10% or 16% (LC-3 only) Tris-glycine gels, and transferred to PVDF membranes. The membranes were blocked in 5% milk and then incubated overnight at 4 °C with primary antibody for AT (1:1000, Diasorin), LC3 (1:2000, Sigma), p62 (1: 1000, Cell signaling, Danvers, MA), ATG5 (1: 1000, Cell signaling), mTOR substrate sampler (1: 1000 for all antibodies, Cell signaling). Donkey anti-goat Ig (Jackson Labs, Bar Harbor, ME), goat anti-rabbit Ig (1:50,000) or goat anti-mouse Ig (1:50,000) were used as secondary antibodies in TBST at RT, 1 hour. Blots were visualized with ECL or West Femto from Pierce. Membranes were re-probed with GAPDH (1: 5000, Sigma) as loading control. Insoluble samples were stained with Gel Code Blue as loading control.

For immunoblot analysis of proteins in liver, the liver was snap frozen in liquid nitrogen and stored at −80°C. Liver was homogenized in 50 mM Tris-HCl pH 8.0, 150mM NaCl, 2 mM KCl, 2 mM MgCl2, 0.5% Triton X-100, 0.5% deoxycholic acid containing 0.1 mM

phenylmethylsulfonic acid and Complete protease inhibitor cocktail from Roche. Total protein concentration was measured by BCA assay (Pierce) and followed by western blotting as described previously.

Radio immunoprecipitation and SDS-PAGE. For the pulse labeling experiments, HTO/Z cells were incubated for 48 hours in the absence or presence of GLB (40 μM) and then subjected to [^{35}S] methionine labeling for 30 mins. The cell lysates were then examined by immunoprecipitation and the immunoprecipitates analyzed by SDS-PAGE/fluorography. For the pulse-chase experiments, HTO/Z cells were incubated for 48h in the absence or presence of GLB (40 μM), then were subjected to pulse radiolabeling for 60 mins and chase in medium with excess unlabeled methionine for time periods up to 300 mins. The extracellular fluid and cell lysate samples were subjected to immunoprecipitation and the immunoprecipitates analyzed by SDS- PAGE/fluorography. All fluorograms were subjected to densitometry. The relative densitometric value of T0 was set at 100% and the remainder of the data set expressed as % of this control. The data are shown as mean +/− SD and the mean value at each time point is shown at the bottom of the figure.

Real Time Quantitative PCR. Total RNA was extracted from PiZ mouse livers or HTO/Z cells with TRIZOL (Life Technologies, Grand Island, NY). First strand cDNA (RT reaction) was synthesized from 2 μg of RNA using high capacity RNA-to-cDNA kit (Applied Biosystems, Foster City, CA) according to the manufacturer's directions. A negative control was performed without enzyme (NRT reaction). RT and NRT reactions were also performed on 2 μg of commercially prepared liver RNA (Ambion, Austin, TX) to serve as the calibrator for the real time QPCRs. Each experimental sample was normalized to a nontransgenic control (fold change). For PCR, duplicate aliquots of the RT reaction and 1 aliquot of the NRT reaction served as templates for the target genes and the control gene β-glucuronidase (GusB). The probes and primers were obtained from Applied Biosystems (SERPINA1 assay ID: Hs01097800-m1; mouse GusB assay ID: Mm00446953-m1; human GusB assay ID: Hs9999908-m1; human MRP1 assay ID: Hs01561483_ml; human MRP3 assay ID: Hs00978452_ml). Real time reactions were run on an ABI7300 using the following cycling parameters: 95°C for 12 min, followed by 40 cycles of 95°C for 15 s and 60°C for 1 min. Differential gene expression was calculated by the ΔΔCT calculation (4). The ΔΔCT method controls for potential differences in efficiency of the RT, as well as the PCR, whereas calculations based on standard curves do not (ΔΔCT = ΔCT(exp) - ΔCT (ctr) when ΔCT = ΔCT(gene) - ΔCT(norm)).

Analysis of experiments in PiZ mouse. The outcome was determined by evaluating 1) the hepatic ATZ load by immunoblotting with anti-AT, immunostaining with PAS/D; 2) hepatic fibrosis using Sirius Red staining; 3) autophagy by immunoblotting with anti-LC3 and anti-p62, and imaging for GFP+ autophagosomes; 4) monitoring blood glucose levels by glucometer and blood insulin levels by ELISA Wide Range Assay (CrystalChem). For immunostaining, liver samples were fixed in 10% Formalin, followed by staining with PAS after diastase and Sirius Red using standard techniques (2). Quantitative evaluation of immunostaining and histochemical staining was carried out by a member of the team that was blinded to group allocation.

Statistical Analysis. Sample size was initially chosen based on previous studies with other drugs that had a statistically significant effect and subsequently was based on results of the initial experiments. No animals, samples, or data points were excluded from the reported analysis once obtained. Student's t-test was used for most comparisons but the Welch modified t-test was used to compare experimental groups that were not paired and did not assume equal variances. Kinetic and dose-response curves were analyzed by two-way ANOVA with the Bonferroni post-test using the Prism software application. All analyses were considered statistically significant at $P<0.05$.

Animal Committee Approvals. All animal experiments were approved by the IACUC at University of Pittsburgh and the ASC of Washington University in St. Louis.

References

1. Fu Y, Foden JA, Khayter C, Maeder ML, Reyon D, Joung JK, (2013). High-frequency off-target mutagenesis induced by CRISPR-Cas nucleases in human cells. Nat. Biotechnol. 2013; 31: 822–826. Doi: 10.1038/nbt.2623. Epub 2013 Jun 23. Doi:10.1038/nbt.2623.

2. Hidvegi T, Ewing M, Hale P, Dippold C, Beckett C, Kemp C, Maurice N, Mukherjee A, Goldbach C, Watkins S, Michalopoulos G, Perlmutter DH (2010). An autophagy-enhancing drug promotes degradation of mutant alpha-1-antitrypsin Z and reduces hepatic fibrosis. Science 329(5988):229-232. Doi: 10.1126/science.1190354. Epub 2010 Jun 3.

3. Hidvegi T, Schmidt BZ, Hale P, Perlmutter DH (2005). Accumulation of mutant alpha1-antitrypsin Z in the endoplasmic reticulum activates caspases-4 and -12, NFkappaB, and BAP31 but not the unfolded protein response. J Biol Chem 280(47):39002-39015. Epub 2005 Sep 23. Doi: 10.1074/jbc.M508652200.

4. Livak KJ, Schmittgen TD (2009). Anylysis of relative gene expression data using real-time quantitative PCR and the $2^{-\Delta\Delta}C_T$ method. Methods 01 Dec 2001, 25(4):402-408.

Figure A.1

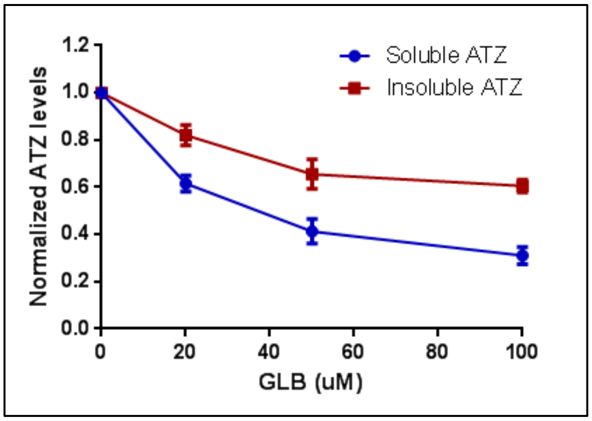

Figure A.2

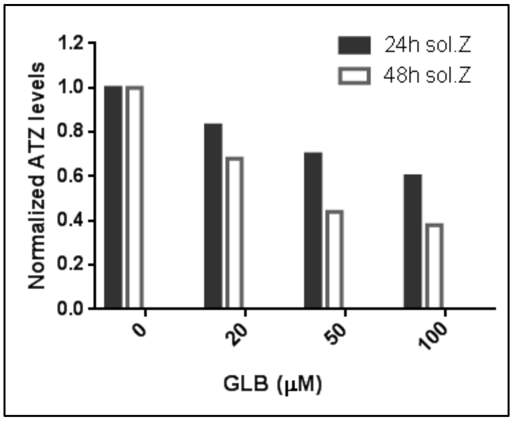

Figure A.3

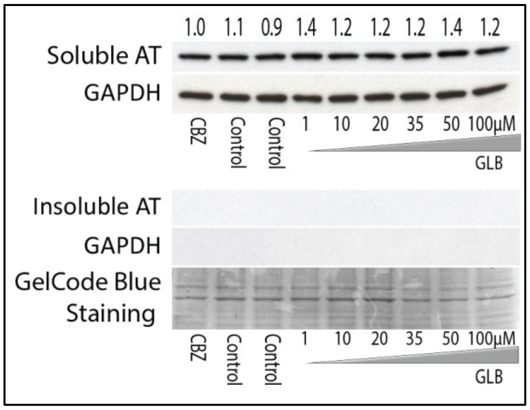

Figure A.4

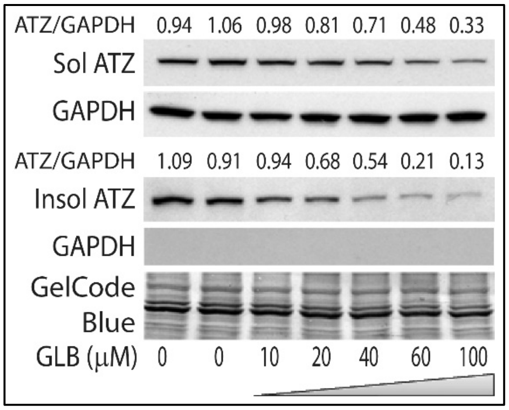

Figure A. Effect of GLB on steady state levels of AT variants in mammalian cell line models. (1) Immunoblot analysis of HTO/Z cells treated with various concentrations of GLB. Quantitative results from 4 independent experiments are shown on the graph. Relative densitometric values of ATZ at each dose are normalized by arbitrarily setting the ATZ levels in DMSO-treated cells at 1.0 (Mean $\pm$ SEM, n=4). The effect of GLB on soluble and insoluble ATZ levels was highly significant, $p<0.0001$ in each case, using 2-way ANOVA. **(2)** Immunoblot analysis of HTO/Z cells treated with GLB for either 24h or 48h. Relative densitometric values of ATZ are normalized in the same way. **(3)** Immunoblot analysis of HTO/Saar cells treated with various concentrations of GLB. **(4)** Immunoblot analysis of HG2TONGZT cells treated with various concentrations of GLB. For **(3)** and **(4)**, after 48-hr incubation in the absence or presence of GLB, cells were harvested and separated into soluble and insoluble fractions for western blotting with anti-AT. GAPDH was used as a loading control for soluble fraction and to validate the separation of soluble and insoluble fractions. Gel Code blue staining was used a loading control for the insoluble fraction. Relative densitometric values for levels of ATZ in **(3)** and ATSaar variant in **(4)** are shown at the top of the gel. Carbamazepine at 30 µM was used as a positive control.

Figure B.1

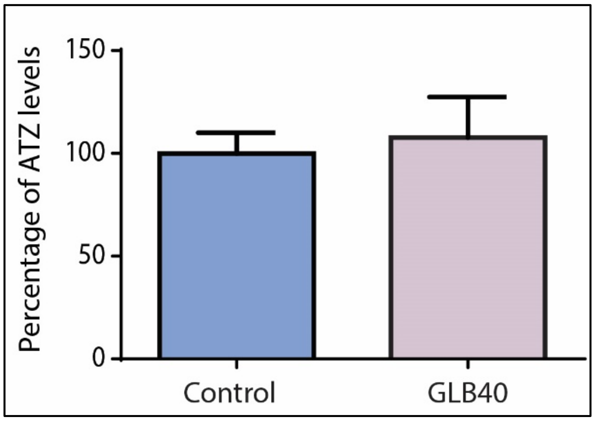

Figure B.2

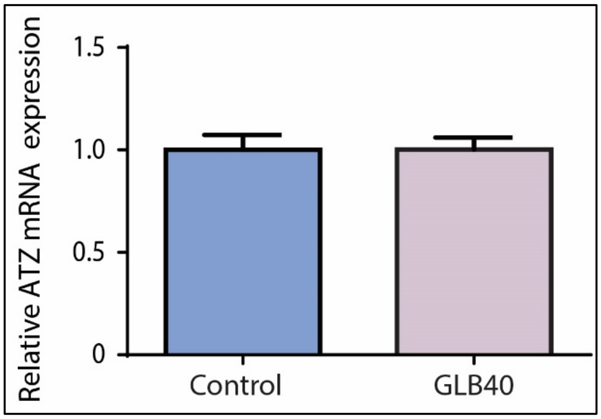

Figure B.3

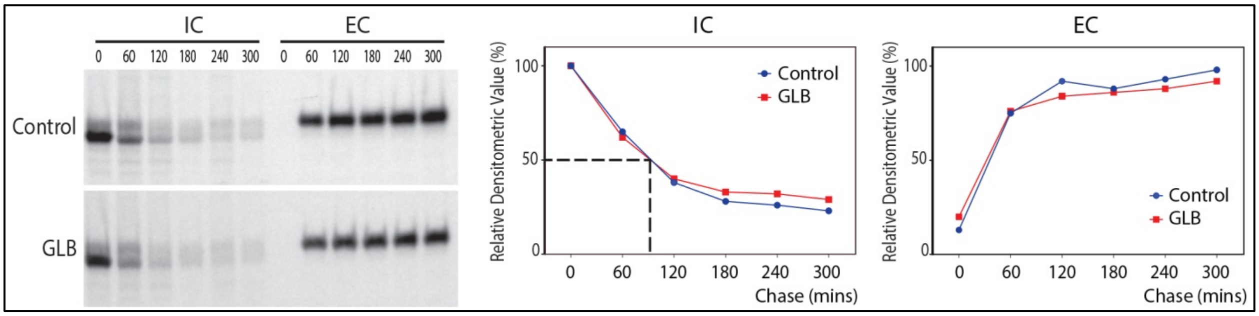

Figure B. Effect of GLB on synthesis of ATZ in HTO/Z cell line (1), RNA levels in HTO/Z cell line (2) and on kinetics of secretion of AT in HTO/M cell line (3). (1) HTO/Z cells were incubated in presence or absence of GLB (40 µM) for 48 h. After pulse labeling, cell lysates were immunoprecipitated with anti-AT. Relative densitometric values were determined with control arbitrarily set at 100% (Mean $\pm$ SD, n=3). Student t-test showed no statistical difference between the GLB-treated group and untreated control cells (p =0.5820, n=3). **(2)** HTO/Z cells were incubated for 48h in presence or absence of GLB (40 µM), and then harvested, lysed and mRNA levels for AT were determined by real-time RT-PCR (Mean $\pm$ SD, n=3). Student t-test showed no statistical difference between the GLB-treated and untreated control cells (p = 0.9757, n=4). **(3)** Left panel shows fluorograms of control (top) and GLB-treated cells (bottom) using the HTO/M cell line. Center panel shows densitometric analysis of kinetics for intracellular (IC) with dashed lines for the half-time for disappearance of wild type AT. Right panel shows densitometric analysis of kinetics for extracellular (EC).

Figure C.1

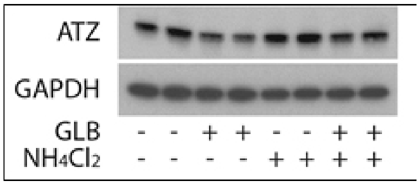

Figure C.2

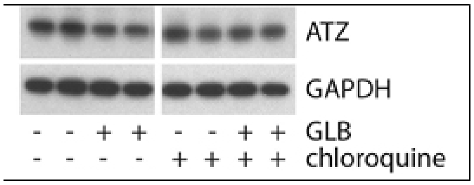

Figure C.3

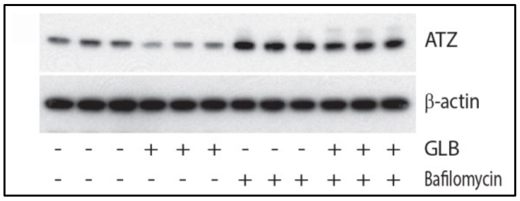

Figure C.4

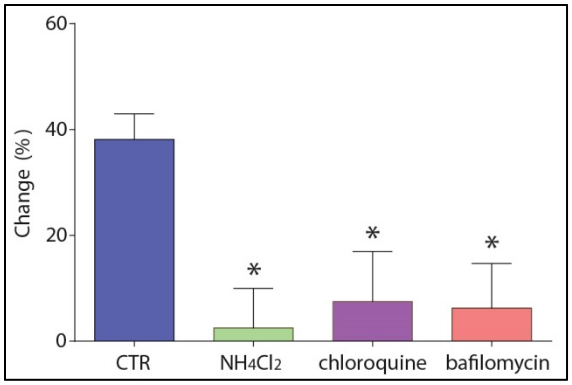

Figure C. Mechanism of ATZ degradation targeted by GLB. **(1)** Effect of GLB on ATZ levels in the HTO/Z cell line in presence or absence of ammonium chloride. **(2)** Effect of GLB on ATZ levels in HTO/Z cell line in the presence or absence of chloroquine. **(3)** Effect of GLB on ATZ levels in the HTO/Z cell line in presence or absence of bafilomycin. Cells were incubated for 48 hours with or without GLB (40 µM) then ammonium chloride (10 mM) or chloroquine (50 µM) or bafilomycin (100 nM) was added for the last 15 hours of this incubation period. **(4)** The relative effect of GLB on ATZ levels in the absence or presence of lysosomal enzyme inhibitors was determined by densitometric scanning. The results are reported here as % change for the effect of GLB. In the absence of lysosomal inhibitor is described as the control (bar labelled CTR, n=16). This is compared to % change for GLB in samples also treated with NH_4Cl_2 (n=4), chloroquine (n=4) or bafilomycin (n=8). *Significant difference compared to CTR ($p<0.05$, unpaired Student T-test).

Figure D.1

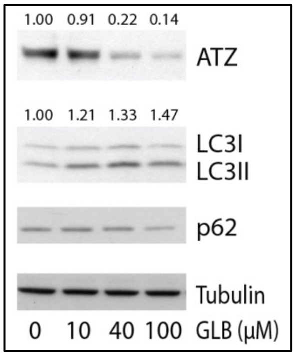

Figure D.2

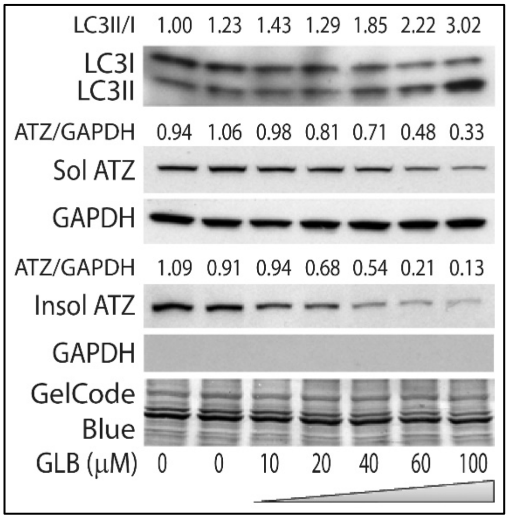

Figure D. Effect of GLB on LC3 conversion and p62 levels. (1) Effect of GLB on LC3 conversion and p62 levels in the HTO/Z cell line by immunoblotting. Densitometric values are shown on top of the blots. GLB doses are shown at the bottom (μM). **(2)** Effect of GLB on ATZ and LC3-II to LC3-I ratio in HG2TONGZT#1 cell line. HG2TONGZT#1 cells were incubated for 48 h in the absence or presence of GLB at various concentrations and then harvested for immunoblot analysis of LC3, AT and GAPDH. GelCode blue staining is shown in the bottom panel. Relative densitometric values are shown at the top of the gels.

Figure E.1

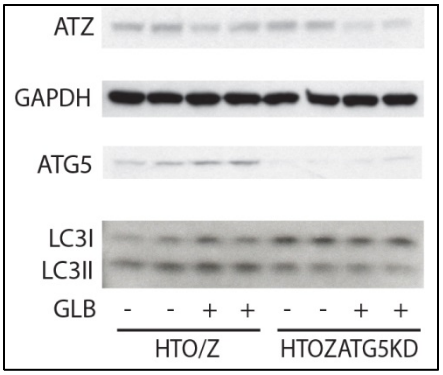

Figure E.2

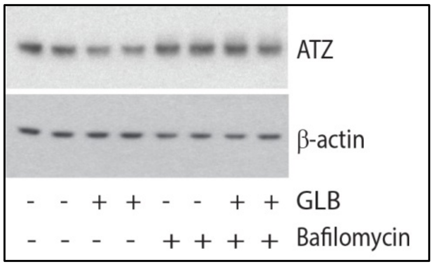

Figure E. Effect of GLB on ATZ levels in the ATG5-deficient HTOZATG5KD cell line.**(1)** HTO/Z and HTOZATG5KD cell lines were incubated in presence or absence of GLB 40 μM for 48 hours. The effect of GLB on ATZ levels was examined by immunoblotting with anti-AT. GAPDH was used as the loading control. ATG5 and LC3 levels were analyzed to verify the decrease in ATG5 levels and in LC3-II to LC3-I ratio. **(2)** HTOZATG5KD cells were incubated for 48 hours in the absence or presence of GLB and bafilomycin 100 nM was added to separate aliquots for the last 15 hours of the incubation period with analysis for ATZ and β-actin levels as the read-out.

Figure F.1

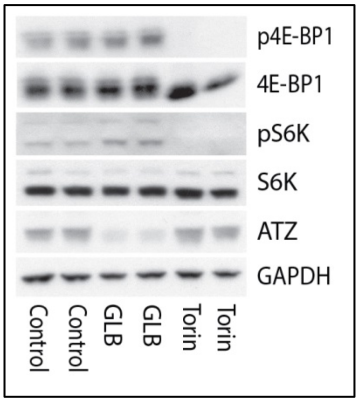

Figure F.2

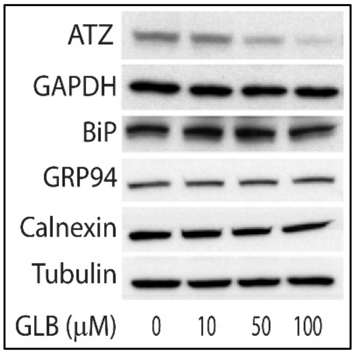

Figure F. Effect of GLB on phosphorylation of TOR substrates (p70, S6K and 4E-BP1) and unfolded protein response in HTO/Z cell line. **(1)** Torin-1 at 250nM was included as a positive control. Total protein and the phosphorylation of p70 S6K and 4E-BP1 were examined by immunoblotting after the HTO/Z cells were treated with GLB or Torin-1 (250 nM). p70 S6K is marked as S6K, phospho-p70 S6K is marked as pS6K; phospho-4E-BP1 is marked as p4E-BP1 on the figure. **(2)** HTO/Z cells were incubated with or without GLB for 48 hrs. and then examined by immunoblotting with antibodies to the UPR proteins, including BiP, GRP94 and calnexin together with tubulin as loading control.

Figure G

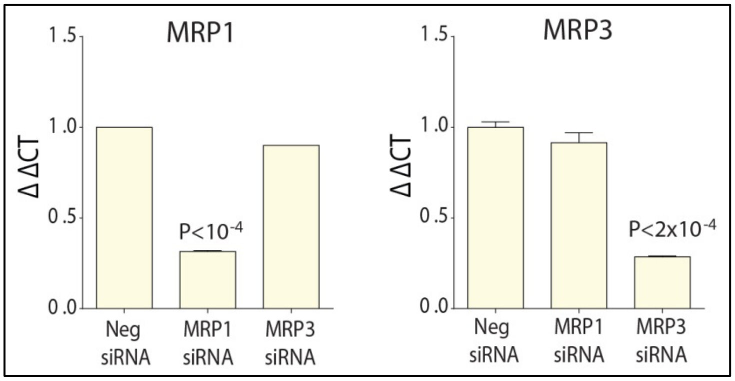

Figure G. Effect of MRP1 and MRP3 and negative control siRNA on MRP1 (left) and MRP3 (right) mRNA levels in HTO/Z cell line. HTO/Z cells were transfected with MRP1 siRNA, MRP3 siRNA or negative control siRNA at a final concentration of 30nM. After transfection, the cells were incubated in the presence or absence of GLB for 48 hours. Quantification of MRP1 and MRP3 mRNA expression by real-time RT-PCR. Results of one-way ANOVA analysis show significant differences as indicated by p values above the appropriate bar in each case (Mean $\pm$ SEM, n=4).

Figure H.1 Figure H.2

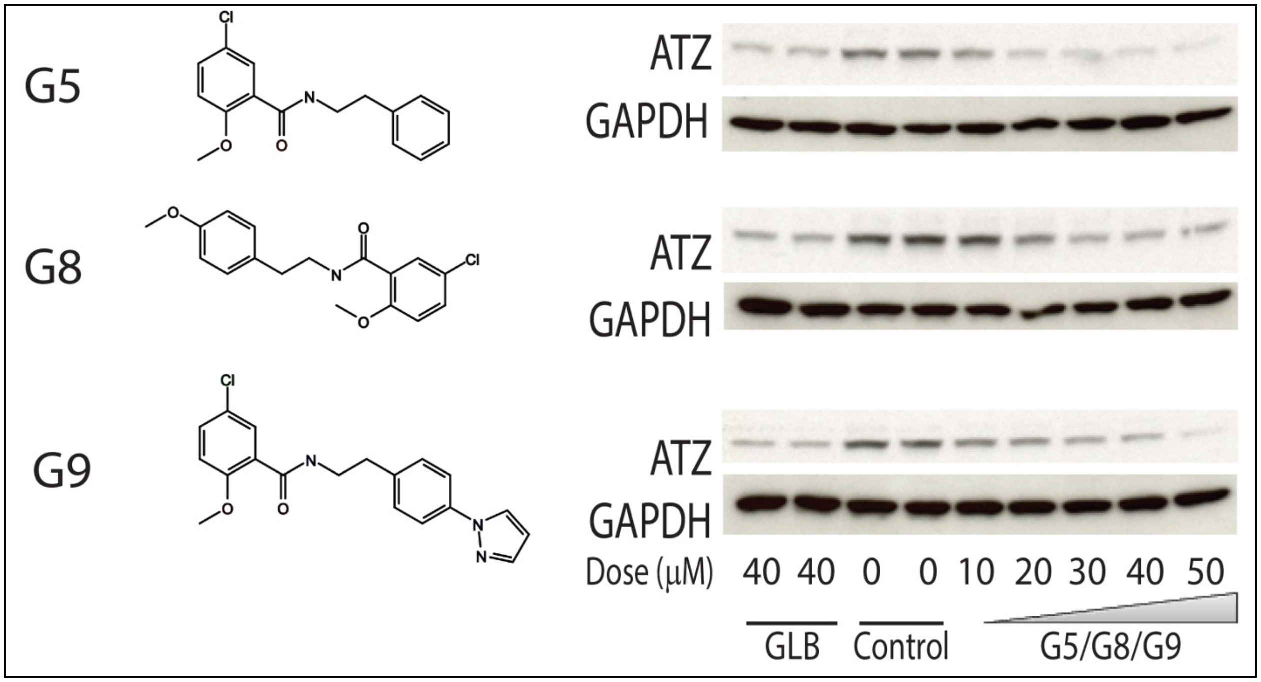

Figure H. Effect of G4 analogs (G5, G8 and G9) on ATZ. (1) The structures of G4 analogs: G5, G8 and G9. **(2)** Immunoblot analysis of the effect of G5 G8 and G9 on ATZ levels in HTO/Z cell line. GAPDH was included as a loading control. GLB was used as positive control; DMSO was used as negative control.

Figure I.1

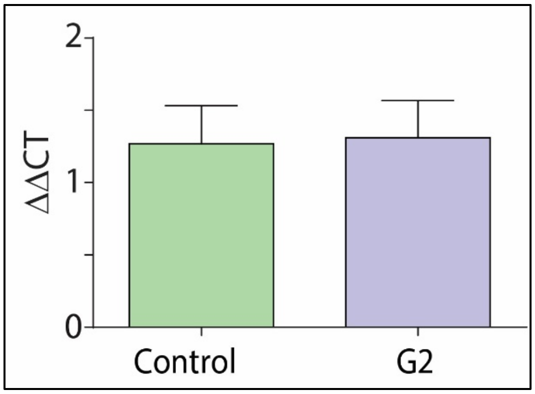

Figure I.2

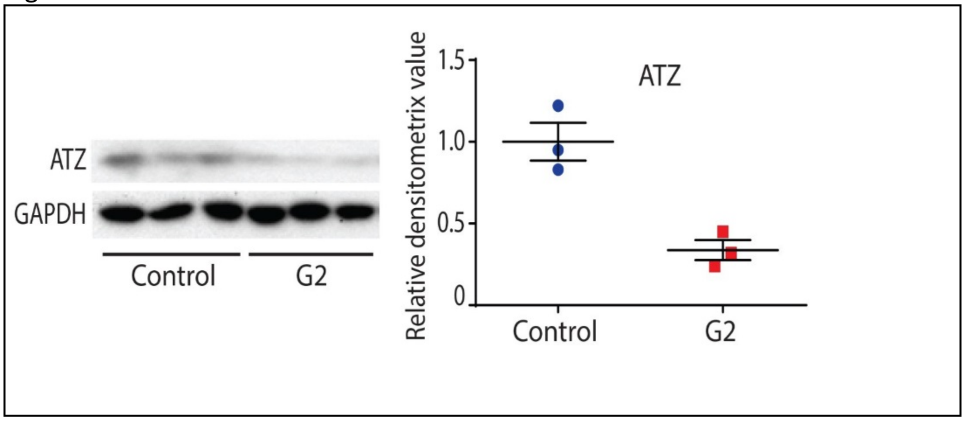

Figure I.3

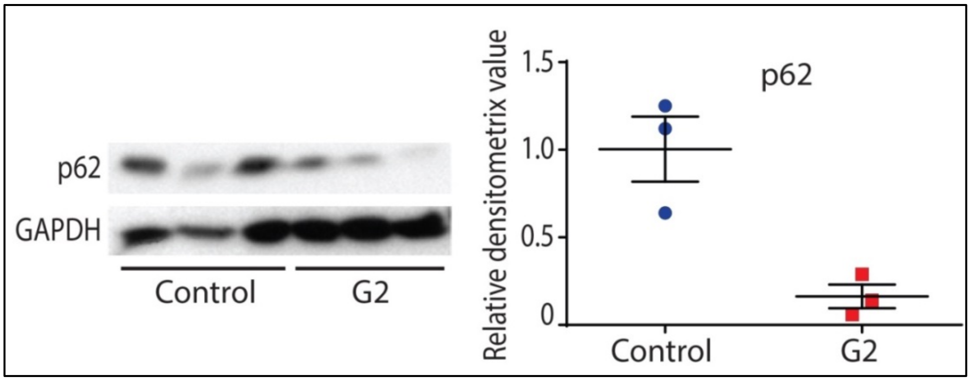

Figure I. In vivo effect of GLB analog G2 on hepatic ATZ and p62 levels. (1) Hepatic ATZ RNA levels by RT-QPCR. Differential gene expression is shown on the vertical axis. No difference was detected (p=0.9137). **(2)**Immunoblot analysis for ATZ (Mean $\pm$ SEM, n=3) levels is shown on left and relative densitometric values on right. The difference was statistically significant (p=0.0257). **(3)** Immunoblot analysis for p62. The difference was significant (p=0.0363). Statistical analysis used unpaired two-tailed t-test.
